# Characterization of a 2016 Clinical Isolate of Zika Virus in Non-human Primates

**DOI:** 10.1016/j.ebiom.2016.09.022

**Published:** 2016-09-23

**Authors:** Xiao-Feng Li, Hao-Long Dong, Xing-Yao Huang, Ye-Feng Qiu, Hong-Jiang Wang, Yong-Qiang Deng, Na-Na Zhang, Qing Ye, Hui Zhao, Zhong-Yu Liu, Hang Fan, Xiao-Ping An, Shi-Hui Sun, Bo Gao, Yun-Zhi Fa, Yi-Gang Tong, Fu-Chun Zhang, George F. Gao, Wu-Chun Cao, Pei-Yong Shi, Cheng-Feng Qin

**Affiliations:** a State Key Laboratory of Pathogen and Biosecurity, Beijing Institute of Microbiology and Epidemiology, Beijing 100071, China; b Laboratory Animal Center, Academy of Military Medical Science, Beijing 100071, China; c Guangxi Medical University, Xining 530021, China; d Guangzhou Eighth People's Hospital, Guangzhou Medical University, Guangzhou, China; e CAS Key Laboratory of Pathogenic Microbiology and Immunology, Institute of Microbiology, Chinese Academy of Sciences, Beijing, China; f Department of Biochemistry and Molecular Biology, Department of Pharmacology and Toxicology, Sealy Center for Structural Biology & Molecular Biophysics, University of Texas Medical Branch, Galveston, TX 77555, USA

**Keywords:** Zika virus, Non-human primate model, Target organ, Lacrimal fluid

## Abstract

Animal models are critical to understand disease and to develop countermeasures for the ongoing epidemics of Zika virus (ZIKV). Here we report a non-human primate model using a 2016 contemporary clinical isolate of ZIKV. Upon subcutaneous inoculation, rhesus macaques developed fever and viremia, with robust excretion of ZIKV RNA in urine, saliva, and lacrimal fluid. Necropsy of two infected animals revealed that systematic infections involving central nervous system and visceral organs were established at the acute phrase. ZIKV initially targeted the intestinal tracts, spleen, and parotid glands, and retained in spleen and lymph nodes till 10 days post infection. ZIKV-specific immune responses were readily induced in all inoculated animals. The non-human primate model described here provides a valuable platform to study ZIKV pathogenesis and to evaluate vaccine and therapeutics.

## Highlights

• Subcutaneous inoculation of ZIKV causes fever and viremia in NHPs
• Robust viral RNA excretion was seen in urine, saliva, and lacrimal fluid.
• The intestinal tracts, spleen, parotid glands, and lymph nodes us are potential target organs.
• ZIKV infection induces robust humoral and cellular immune response.

Zika virus (ZIKV) is spreading throughout the world whereas no vaccine or drug is currently available. To better understand the disease, we established the non-human primate model with a ZIKV strain isolated in 2016. We found that ZIKV infection in Rhesus monkeys resulted in fever, and viral RNA were present in various body fluids, including blood, urine, saliva, and lacrimal fluids. We identified the intestinal tracts, spleen, parotid glands, and lymph nodes as potential target organs of ZIKV by necropsy. The animal model not only helps understand the disease, but also provides a powerful tool for vaccine and antiviral tests.

## 1 Introduction

Zika virus (ZIKV) used to be an obscure mosquito-borne flavivirus from *Flavivirus* genus within *Flaviviridae* family. Other flaviviruses of global importance include dengue virus (DENV), West Nile virus (WNV), yellow fever virus (YFV), Japanese encephalitis virus (JEV), and tick-borne encephalitis virus (TBEV). ZIKV is phylogenetically divided into two lineages: the African and Asian lineages (Haddow et al., 2012). Since 2007, the Asian lineage of ZIKV has caused epidemics in Polynesia, the South Pacific, and most recently the Americas, leading to global concerns about its association with microcephaly and severe neurologic disorders (Gulland, 2016). The causal linkage between ZIKV infection and microcephaly, initially indicated by clinical studies, has recently been recapitulated in mouse models. ZIKV can infect mouse fetus, resulting in intrauterine growth restriction, placental damage, microcephaly, and fetal demise (Cugola et al., 2016, Li et al., 2016, Miner et al., 2016, Wu et al., 2016).

Despite the above progress, the pathogenesis and transmission of ZIKV remain largely unknown. Recent data suggested human dermal fibroblasts, epidermal keratinocytes, placental macrophages and neural progenitor cells were permissive to ZIKV infection (Hamel et al., 2015, Li et al., 2016, Quicke et al., 2016, Tang et al., 2016). Results from mouse model suggest that ZIKV replicates efficiently in embryonic mouse brain by directly targeting neural progenitor cells and causing apoptosis (Cugola et al., 2016, Li et al., 2016). In patients, infectious ZIKV particles have been detected in blood, urine (Zhang et al., 2016), saliva (Barzon et al., 2016), and breastmilk (Dupont-Rouzeyrol et al., 2016). There is increasing evidence of sexual transmission of ZIKV (D'Ortenzio et al., 2016, Moreira et al., 2016), and ZIKV RNA and infectious particles have been detected in semen in ZIKV-infected patients (Atkinson et al., 2016, Mansuy et al., 2016) or testis in infected mice (Lazear et al., 2016, Miner et al., 2016). However, due to the highly correlated nature of sexual behaviors, sexual and close contact transmission by saliva or other body fluids can be difficult to distinguish, whether such unusual viral excretions contribute to non-mosquito-mediated transmission remains to be determined. The knowledge of in vivo replication, excretion kinetics, and target tissues/organs of ZIKV is urgently needed for understanding the disease and pathogenesis.

No vaccines and antiviral drugs are currently available to prevent and treat ZIKV infection. Animal models are essential for the development of such countermeasures. Young A129 mice (lacking interferon α/β receptor) and AG129 (lacking interferon α/β and γ receptors) were recently reported to succumb to ZIKV infection and to develop neurological signs (Aliota et al., 2016, Lazear et al., 2016, Malone et al., 2016). Since these mouse models are deficient in innate immune response, an immune competent animal model is needed. Non-human primates have been well documented as a more relevant animal model for flavivirus infections (Sariol and White, 2014, Zompi and Harris, 2012), and have been widely used for DENV and WNV pathogenesis studies and vaccine efficacy tests (Sariol and White, 2014). ZIKV was first isolated from a febrile rhesus macaques (Dick et al., 1952). Multiple monkey species in forests were found to be seropositive for ZIKV (McCrae and Kirya, 1982), suggesting that non-human primates can be infected and support viral replication.

Initial experiments performed in 1950s showed that rhesus monkeys inoculated subcutaneously (s.c.) or intracerebrally (i.c.) with the African ZIKV strain MR766 developed no signs of pyrexia, but generated antibodies within 2 to 3 weeks after infection (Dick, 1952). However, bioinformatics analysis suggests that the ongoing epidemic strains in the Americas have accumulated some amino acid changes that might contribute to the explosive epidemics (Faria et al., 2016, Wang et al., 2016). Here, we have established a non-human primate model using a contemporary ZIKV strain GZ01/2016 (GenBank accession no: KU820898) that was isolated from a patient returned from Venezuela to China in 2016 (Zhang et al., 2016). ZIKV infection upon subcutaneous in rhesus macaques resulted in fever, viremia, and robust viral shedding in multiple body fluids including saliva, urine, and lacrimal fluids. The major target organs of ZIKV and specific immune response in non-human primates were also characterized in detail. Our study establishes the non-human primate model of ZIKV infection with contemporary clinical isolate that will be valuable for evaluating candidate vaccines and therapeutics as well as understanding ZIKV pathogenesis, dissemination and transmission.

## 2 Materials & Methods

### 2.1 Ethics Statement

All animal experiments were performed in strict accordance with the guidelines of the Chinese Regulations of Laboratory Animals (Ministry of Science and Technology of People's Republic of China) and Laboratory Animal-Requirements of Environment and Housing Facilities (GB 14925-2010, National Laboratory Animal Standardization Technical Committee). All procedures were performed under sodium pentobarbital anesthesia by trained technicians and all efforts were made to ameliorate the welfare and to minimize animal suffering in accordance with the “Weather all report for the use of non-human primates” recommendations. The experimental protocols were approved by the Animal Experiment Committee of Laboratory Animal Center, AMMS, China (IACUC-13-2016-001).

### 2.2 Viruses and Cells

ZIKV strain GZ01/2016 (GenBank number KU820898) was isolated from a Chinese male patient returned from Venezuela (Zhang et al., 2016). ZIKV stocks were propagated in *Aedes albopictus* C6/36 cells and titrated by plaque forming assay on BHK-21 cells (Dai et al., 2016, Deng et al., 2016). Studies with infectious ZIKV were conducted under biosafety level 2 (BSL-2) facilities at Beijing Institute of Microbiology and Epidemiology.

### 2.3 Study Design

This study was designed to establish the infectivity and viral dynamics of currently circulating Asian lineage ZIKV. Five 5-year-old rhesus monkeys (weighing 5.0 to 6.0 kg) in good health were prescreened negative for IgG antibodies against flaviviruses (including ZIKV, DENV, JEV and YFV) by enzyme-linked immunosorbent assay (ELISA). All the animals were housed individually in a single cage in pathogen-free facility, and acclimatized for a week. Rhesus monkeys were s.c. inoculated with 10^5^ PFU (equivalent to 10^8.5^ RNA copies) of ZIKV. After the inoculation, clinical signs were recorded during 34-day observation period. Blood and major body fluids were collected for determination of viral load. Two rhesus monkeys were euthanasia to perform hematoxylin and eosin (HE) and immunohistochemistry as well as virological analysis for evaluation of viral tropism.

### 2.4 Body Fluids Collection

Urine was collected from a container under the animal's cage, and was stored at − 80 °C until analysis. Saliva was obtained by running a sterile swab under the animal's tongue. Lacrimal fluid was collected by gently running a sterile swab on the animal's eye. Cerebrospinal fluid (CSF) was collected by lumbar puncture at the time of necropsy. Swabs were placed immediately into 1.0 ml of viral transport medium (tissue culture medium 199 supplemented with 0.5% FBS and 1% antibiotic/antimycotic) for 60 min. Samples were vortexed vigorously, then centrifuged for 10 min at 3000 rpm before removing the swabs. Samples were stored at − 80 °C until processing.

### 2.5 Necropsy

Two s.c. inoculated rhesus monkeys were euthanized at days 5 and 10 p.i. by infusion of pentobarbital, and necropsy was performed. Samples were collected from the following tissues: cerebrum, cerebellum, brain stem, spinal cord, lacrimal gland, parotid gland, lung, liver, spleen, stomach, left and right kidneys, small intestine, large intestine, cecum, prostate, epididymis, pancreas, adrenal gland, inguinal lymph nodes, heart, urinary bladder, testis and muscle. All samples were snap frozen for ZIKV-RNA detection and PFU assay. In brief, 1 ml of DMEM with 2% FBS and penicillin/streptomycin was placed in an Eppendorf tube. The organ was weighed and placed in the tube. Tissues were homogenized for 30 s. The homogenate was clarified by centrifugation for 5 min at 5000 rpm. The supernatants of tissues were collected for RNA extraction and virus isolation.

### 2.6 Histology and Immunohistochemistry

Collected tissues from inoculated monkeys were fixed with perfusion fixative (formaldehyde) for 48 h, and processed according to standard histological assays. All sections from each tissue were stained with hematoxylin and eosin (H&E). For immunohistochemistry, sections were deparaffinized with xylene, rehydrated through successive bathes of ethanol/water (from 100% ethanol successively to 50% ethanol till pure water) and incubated in 3% H_2_O_2_ at room temperature. The sections were then put in 10 mM sodium citrate buffer for 1 h at 96 °C for antigen retrieval and blocked with BSA at saturation for 20 min. Monoclonal mouse anti-ZIKV E protein antibody 2A10G6 (Dai et al., 2016) was applied to the section overnight at 4 °C. Horse radish peroxidase (HRP)-conjugated anti-human polyclonal antibody was used as a secondary antibody and 3′-diaminobenzidine as a chromogen. The section was counterstained with hematoxylin.

### 2.7 Viral RNA Extraction and Viral Isolation

Viral RNA was extracted from 0.1 ml of serum or 0.2 ml of urine, saliva and lacrimal fluid samples as well as supernatants of tissues using a PureLink RNA minikit (Life Technology, USA) according to the manufacturer's instructions. RNA was eluted in 60 μl of RNase-free water, aliquoted, and stored at − 80 °C until use.

For virus isolation, virus titers of the selected tissues (Threshold cycle (Ct) values < 30) were determined by plaque forming assay on BHK-21 cells. Briefly, BHK-21 cells in 12-well plates were infected with a 10-fold serially diluted suspension. The plates were incubated at 37 °C for 1 h with gentle rocking every 15 min. The supernatant was removed, and cells were overlaid with 1% low-melting-point agarose (Promega) in DMEM containing 2% FBS. After further incubation at 37 °C for 3–4 days, the cells were fixed with 4% formaldehyde and stained with 0.2% crystal violet to visualize the plaques.

### 2.8 ZIKV RNA Quantification by qRT-PCR

The *Xho* I-linearized plasmid containing full-length genomic cDNA clone of ZIKV strain GZ01/2016 was subjected to in vitro transcription using RiboMAX Large Scale RNA Production System (Promega). The RNA transcript was purified using PureLink RNA minikit (Life Technology) according to the manufacturer's instructions and quantified using spectrophotometry on Nanodrop®. The purified RNA was diluted 10-fold serially using RNases-free water and was detected using quantitative real-time reverse transcriptase PCR (qRT-PCR). Threshold cycle (Ct) values for the known concentrations of the RNA were plotted against the log of the number of genome equivalent copies. The resultant standard curve was used to determine the number of genome equivalents of ZIKV RNA in samples. The determination of the detection limit was based on the lowest level at which viral RNA was detected and remained within the range of linearity of a standard curve. (Ct values< 38.5). qRT-PCR was performed using One Step PrimeScript™ RT-PCR Kit (Takara, Japan) with the primers and probe (Table S1) as described previously (Li et al., 2016). The 20 μl reaction mixtures were set up with 2 μl of RNA. Cycling conditions were as follows: 42 °C for 5 min, 95 °C for 10 s, followed by 40 cycles of 95 °C for 5 s and 60 °C for 20 s.

### 2.9 Detection of ZIKV Strand Specific RNA by Quantitative PCR

For quantification of ZIKV genomic RNA of both positive- and negative-strands in specific organs with high viral RNA loads (Ct values < 30), the 5′-tagged forward (ZIKV-ASF-Tag) and reverse (ZIKV-ASR-Tag) primers (Table S1) were used to obtain cDNA from the negative-(-RNA) and positive-strand RNA (+ RNA), respectively (Plaskon et al., 2009). Briefly, cDNA was synthesized with Superscript II (Invitrogen) at 50 °C for 30 min, and then heat inactivated at 95 °C for 15 min. Quantitative PCR (qPCR) was then performed with the specific primers and probe for strand specific RNA detection. The 20 μl reaction mixtures were set up with 5 μl of cDNA. Cycling conditions were as follows: 95 °C for 10 s, followed by 40 cycles of 95 °C for 5 s and 60 °C for 20 s. The copy numbers of viral RNAs were quantitated as above mentioned.

### 2.10 Antibody Response Assays

Serum IgM and IgG antibodies against ZIKV were detected by ELISA using formaldehyde-inactivated ZIKV. Briefly, 96-well microtiter plates were coated overnight with 10^3^ PFU of inactivated ZIKV. Diluted plasma samples (1:50) were incubated with the coated antigen for 2 h, followed by 1 h incubation of either HRP-conjugated goat-anti-monkey IgM or IgG (Abcam, UK). After washing, TMB-substrate (Promega, USA) was added to the wells and the plate was incubated for 10 min at room temperature in darkness. Then, 2.0 M H_2_SO_4_ was added to stop the reaction, and plates were measured at 450 nm using a microplate reader (Beckman, USA). Endpoint titers were considered the highest dilution that resulted in a value two-fold greater than the absorption of the control serum, with a cut-off value of 0.05.

Neutralizing antibody titers were determined by a constant virus-serum dilution 50% plaque reduction neutralization test (PRNT_50_) as previously described. Briefly, serial 2-fold dilutions of inactivated serum were mixed with equal volumes of ZIKV in DMEM supplemented with 2% FBS. After incubation at 37 °C for 1 h, virus-antibody mixtures were added to plates containing BHK-21 cells. The concentration of infectious virus was determined using the plaque assay described above. The endpoint neutralization titer was calculated according to the method of Reed and Muench.

### 2.11 Biochemistry and Hematology Analysis

A panel of hematological parameters i.e. white blood cell count (WBC), red blood cell count (RBC), hemoglobin (HGB), platelets (PLT), lymphocytes (LYM), monocytes (MON) and neutrophils (NEUT) were analyzed in peripheral blood using a Celltac E MEK-7222 hematology analyzers (Nihon Kohden, Japan). Biochemical analysis, i.e. alanine amino-transferase (ALT), aspartate aminotransferase (AST), total protein (TP), albumin (ALB), glucose GLU, urea (UREA) and creatinine (CREA) was assessed using a 7100 automated biochemical analyzer (Hitachi, Japan).

### 2.12 Cellular Immune Response

Cellular immune responses were assessed in PBMC by the gamma interferon (IFN-γ), interleukin 2 (IL-2) or IL-10 enzyme-linked immunosorbent spot (ELISPOT) human set (Abcam, UK) according to the manufacturer's protocol. Briefly, PBMCs were thawed and washed with Hanks balanced salt solution (HBSS). The cells were then centrifuged at room temperature at 2500 rpm for 15 min without braking, followed by two washes with HBSS. Then, the cells were resuspended in RPMI 1640 containing 10% FBS and diluted to a working concentration of 5 million cells per ml. For ELISPOT analysis, 0.5 or 0.1 million cells per well were seeded with the appropriate antigen stimulation in 96-well tissue culture dishes coated with 5 mg/ml of IFN-γ, IL-2 or IL-10 capture monoclonal antibody. Nonstimulated and PMA (Sigma)-stimulated cells were used as negative and positive controls, respectively. The cells were then cultured for 20 h at 37 °C and 5% CO_2_. Plates were washed, and biotinylated anti-human IFN-γ, IL-2 or IL-10 antibody was added to each well and incubated for 2 h at room temperature. Thereafter, the plates were washed and incubated for 1 h at room temperature with streptavidin- horseradish peroxidase (streptavidin-HRP). Finally, AEC substrate solution (Abcam, UK) was added and spots were counted with an ELISPOT Analysis System (At-Spot-2100, China). Assay results are expressed as the value obtained by the following: (number of spots in experimental well-number of spots in medium control) / 10^6^ cells.

### 2.13 ZIKV Genome Sequencing and Detection of the SNP Sites

Viral RNA was isolated using a PureLink RNA minikit (Life Technology, USA) according to the manufacturer's instructions. The genome cDNA was obtained by reverse transcription (RT) using SuperScript III (Life Technology, USA). For determination of consensus sequence, PCR products were directly sequenced by Sanger sequencing in both directions using virus-specific primers (Table S1). Sequence fragments were assembled into a consensus sequence with DNASTAR software, version 7.0.

High throughput sequencing was performed on an Illumina MiSeq sequencing machine. The genome sequences of the viruses were assembled by mapping the reads to the reference genome of ZIKV strain GZ01/2016. Genomic mapping and single nucleotide polymorphism (SNP) detection were processed with CLC Genomic Workbench. The site with substitution frequency above 5% was considered as a single nucleotide variant (iSNV).

## 3 Results

### 3.1 ZIKV Causes Fever and Viremia in Rhesus Macaques

**Fig. 1:**
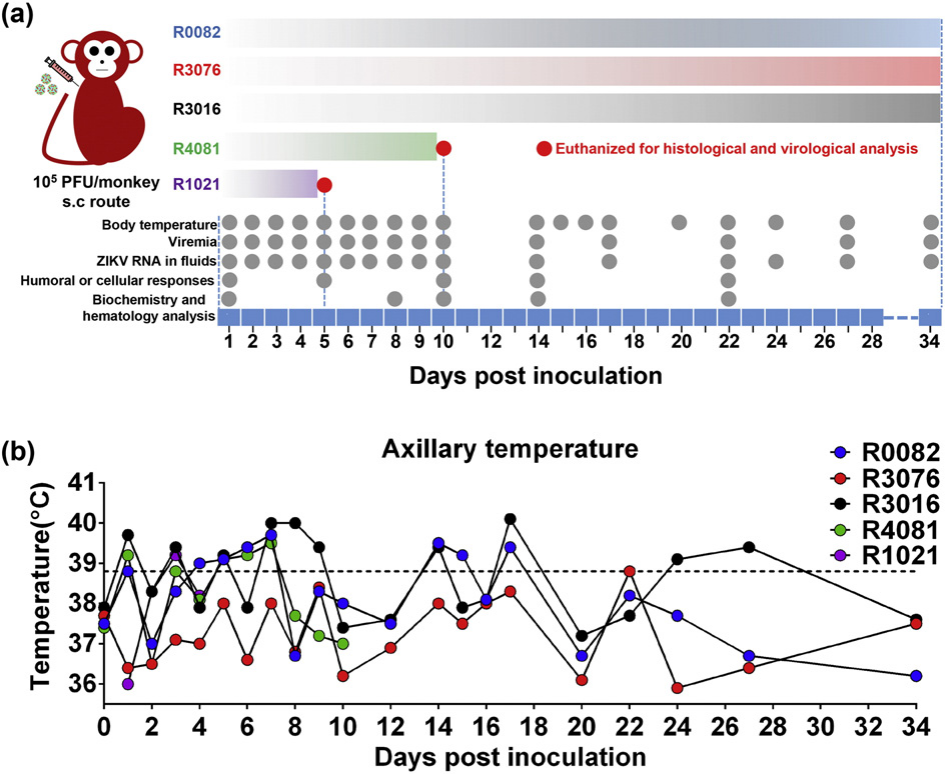
Study design and body temperature change in ZIKV-infected rhesus monkeys. (a) Study design and experimental parameters. Five rhesus monkeys were s.c. inoculated with 10^5^ PFU of ZIKV strain GZ01/2016. Disease parameters were measured including body temperature, blood cell count, and blood chemistry. Viral loads in blood and major body fluids were monitored to evaluate viral kinetics in monkeys. Grey balls indicate detection time points. (b) Change in axillary temperature of rhesus monkeys after s.c. infection with ZIKV. Dotted line indicates the temperature (38.9 °C) for fever determination.

In the present study, to mimic the natural mosquito-biting route of ZIKV, five adult rhesus macaques aged five years old were s.c. inoculated with 10^5^ PFU of ZIKV strain GZ01/2016. Fig. 1a outlines the timeline for clinical manifestations, sample collection, and necropsies. Fig. S1 summarizes the experimental results from each animal. Within 10 days post-infection (p.i.), four out of five animals displayed fever (axillary temperature > 38.9 °C) with peak temperature of 40.1 °C (Fig. 1b). The forehead temperature also increased upon infection in all inoculated monkeys (Fig. S2). Interestingly, fluctuant fever was observed in two animals between days 17 and 27 p.i. (Fig. 1b). No additional signs (e.g., rash and hyperemia) or behavioral abnormalities (e.g., diarrhea, inappetence, dehydration, depression, inactivity, self-injurious, or stereotypical behavior) were observed throughout the experimental period.

Blood chemistry analysis and complete blood cell counts were performed at the indicated times. High levels of liver enzyme alanine aminotransferase (ALT) and aspartate aminotransferase (AST) were observed (Fig. S3a). These enzymes increased significantly at days 1 and 8 p.i., suggesting that liver dysfunction might be an early sign of infection. In addition, a sharp increase in creatinine (CREA) level was detected in two ZIKV-infected animals (Fig. S3a). Some minor changes were observed in blood parameters in several animals, but the values remained within normal variation ranges (Fig. S3b).

**Fig. 2:**
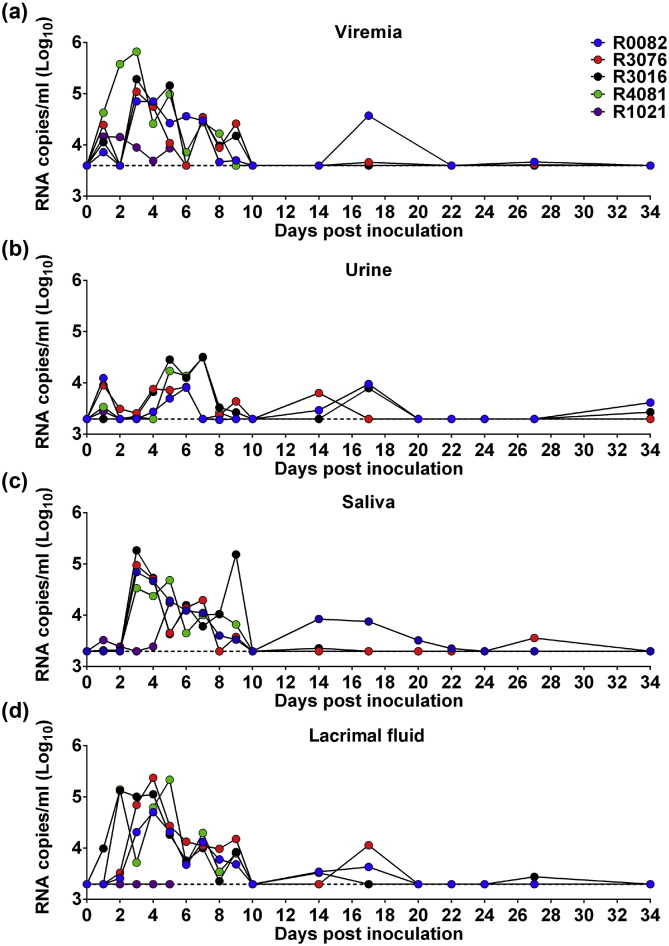
Excretion of viral RNAs in ZIKV-infected rhesus monkeys. (a) Blood. (b) Urine. (c) Saliva. (d) Lacrimal fluid. Dotted lines indicate the limit of detection.

Viremia is well documented as the marker for flaviviral replication in vivo. Blood was sampled daily for 10 days p.i. and every 3–5 days thereafter (Fig. 1a). Plasma viremia was detected by ZIKV-specific qRT-PCR in all tested animals. All animals developed viremia, and the mean viremia duration was 6.6 days. Peak plasma viremia occurred between days 2 and 5 p.i., and ranged from 10^4.16^ to 10^5.82^ RNA copies/mL (Fig. 2a), which is equivalent to those observed in patients (Lanciotti et al., 2008). By day 10 p.i., viremia was undetectable in all animals; However, low level of plasma viral RNA (10^4.57^ RNA copies/mL) re-emerged in two animals (R0082 and R3076) on days 17 and 27 p.i. (Fig. 2a). Taken together, the currently circulating ZIKV strain can cause fever and viremia in adult rhesus macaques, which partly recapitulates the clinical manifestations of ZIKV infection in humans.

### 3.2 ZIKV Viral RNA Excretion in Various Body Fluids

Viral shedding in body fluids is critical for both disease diagnosis and virus transmission, we then assayed the viral RNA kinetics in various body fluids. Remarkably, ZIKV RNA could be readily detected in urine of all infected animals on day 1 p.i., and the levels of viral RNA peaked on days 5 to 7 with 10^4.51^ RNA copies/ml. The mean duration time was 7.5 days. Prolonged viral RNA shedding was observed in two animals on days 14 and 17 p.i., and low levels of ZIKV RNA re-appeared in urine after one month in two animals (Fig. 2b). In saliva, viral RNA could be detected on day 3 p.i. and afterwards, and peaked at 3–9 days p.i. Prolonged shedding of viral RNA in saliva was also observed in two animals (Fig. 2c). These observations are in agreement with clinical results that ZIKV RNA was detected in urine and saliva (Barzon et al., 2016, Gourinat et al., 2015, Roze et al., 2016).

Unexpectedly, robust viral RNA shedding was detected in lacrimal fluid in four of the five animals from days 1 to 10 p.i., and peaked at 2–5 days p.i. Especially, viral RNA was detected in two macaques on day 17 p.i. (Fig. 2d). Ocular complications have been recently described in ZIKV patients (de Paula Freitas et al., 2016, Ventura et al., 2016a), here demonstrated the excretion of ZIKV RNAs in lacrimal fluid in ZIKV-infected animals. Additionally, ZIKV RNA was detected in the cerebrospinal fluid (CSF) on day 5 p.i. (Fig. S1). Overall, ZIKV RNA shedding was detected in multiple body fluids, and it is notable that viral RNA levels were different among distinct body fluids, in the order of blood > lacrimal fluid ≥ saliva > urine.

### 3.3 ZIKV Targets Various Organs and Causes Pathological Damage

**Fig. 3:**
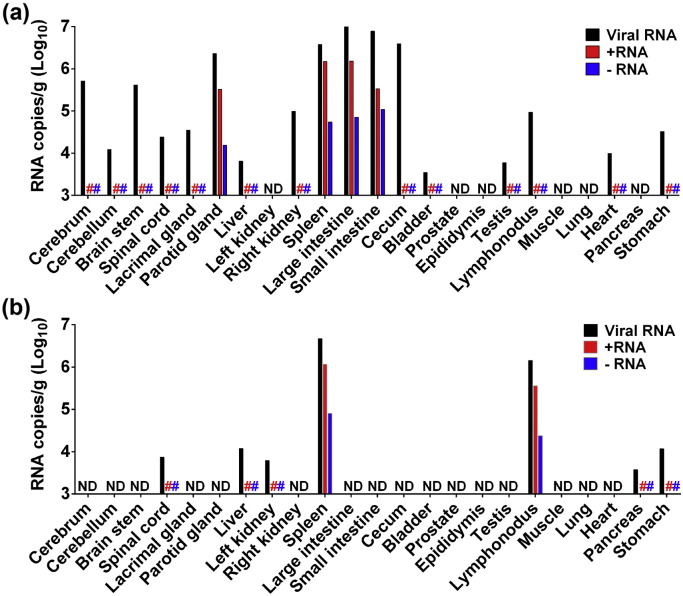
Detection of viral RNA in tissues of ZIKV-infected rhesus monkeys. Viral RNA, + RNA (positive-strand viral RNA) and − RNA (negative-strand viral RNA) were determined in tissues of ZIKV-challenged rhesus monkeys on day 5 (a) and day 10 (b) p.i. The corresponding primers and probes were listed in Table S1. ND, not detectable. #, not done.

To verify the in vivo target organs and the pathological changes caused by ZIKV infection, we euthanized two animals and performed necropsies at days 5 and 10 p.i., respectively. On day 5 p.i., ZIKV RNA was detected in both central nervous system (CNS; including cerebrum, cerebellum, brain stem, and spinal cord), and visceral organs (including liver, kidney, spleen, parotid glands, large intestine, small intestine, cecum, bladder, testes, lymph node, heart, and stomach). The highest level of viral RNA was detected in large intestine, small intestine, cecum, spleen and parotid glands (Fig. 3a). The intestines have also been documented as potential target organs of DENV infection in mice (Schoggins et al., 2012). Interestingly, on day 10 p.i., viral RNA was cleared in most tissues except for spinal cord, spleen, lymph node, liver, kidney, pancreas, and stomach (Fig. 3b). Although samples were collected from two animals, viral RNAs in spleen and lymph nodes were higher than that on day 10 p.i. compared with that on day 5 p.i., suggesting potential active viral replication in these tissues at later stage of ZIKV infection. Significantly, negative-strand ZIKV RNA was detected in spleen, parotid glands, large intestine, small intestine, and cecum on day 5 p.i.,(Fig. 3a) and on day 10 p.i. spleen and lymph nodes still tested positive for negative-strand ZIKV RNA (Fig. 3b). The level of negative-stand RNA in each tested organ was much lower than that of positive-strand RNA as expected (Table S2). Infectious ZIKV was also directly recovered from the above-mentioned target organs with high viral load. Together, all these results demonstrated active ZIKV replication in these organs.

**Fig. 4:**
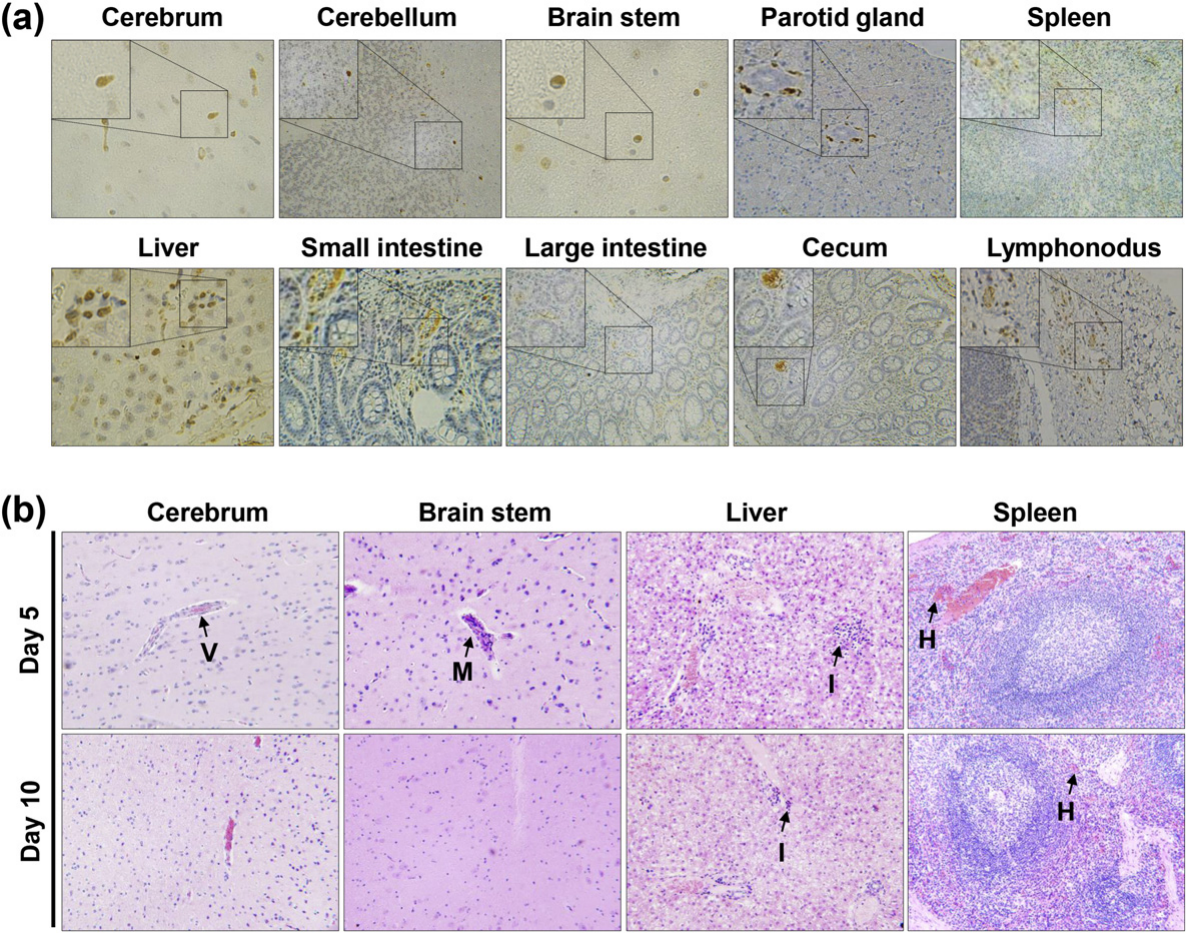
Immunohistochemistry and histopathological changes of tissues of inoculated rhesus monkeys. (a) Immunohistochemistry of tissues of inoculated rhesus monkey on day 5 p.i. Samples were stained with the pan-flavivirus mouse monoclonal antibody 2A10G6. Brown colored staining suggests ZIKV infection. (b) Histopathological changes of tissues of inoculated rhesus monkeys on days 5 and 10 p.i. Arrows denote vascular cuffing (V), mononuclear inflammatory cell infiltration (M), inflammatory cell infiltration (I), and hemorrhage (H).

Immunohistochemistry using a pan-flavivirus mouse monoclonal antibody 2A10G6 (Dai et al., 2016) revealed that ZIKV-specific antigens were present in multiple CNS and peripheral organs during the acute phase of infection (Fig. 4a), which was in agreement with the results of viral RNA detection (Fig. 3). Histopathological examination showed substantial pathological changes in cerebrum, cerebellum, brain stem, liver, and spleen on day 5 p.i., characterized by vascular cuffing in cerebrum and brain stem, inflammatory cell infiltration in liver, and hemorrhage in spleen (Fig. 4b). Liver damage has been previously described in ZIKV patients (Macnamara, 1954), and is potentially relevant to the elevated levels of AST and ALT observed in our study (Fig. S3a). Collectively, these above observations suggested that (i) ZIKV established systematic infections involving both CNS and visceral organs at the early stage; (ii) intestinal tracts, spleen, and parotid glands are the major target organs of ZIKV; (iii) lymph nodes and spleen support active ZIKV replication when viremia and clinical symptoms have disappeared.

**Fig. 5:**
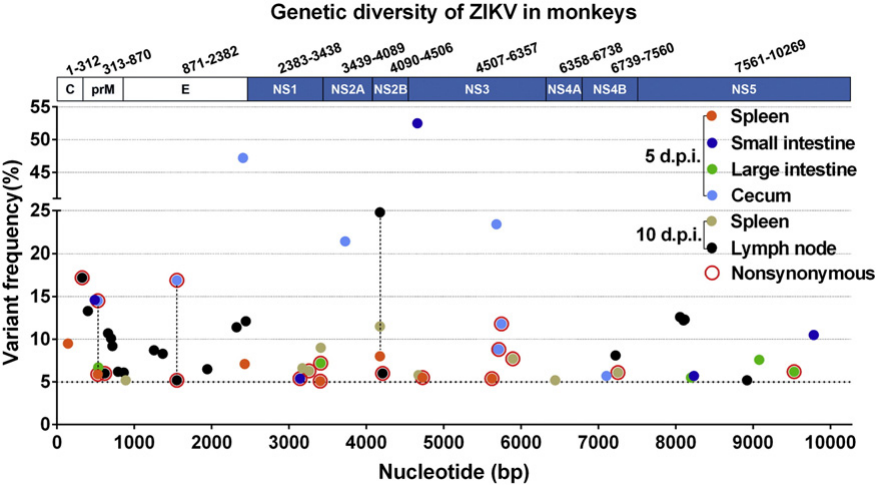
Genetic diversity of ZIKV in various tissues from the rhesus monkey. Consensus changes found in tested tissues were connected with dotted lines.

Additionally, routine viral genome sequencing of samples from the selected organs failed to identify any adaptive mutation in all tested samples. High-throughput sequencing was then performed by using an Illumina MiSeq sequencing machine. Genomic alignment revealed a panel of intrahost single nucleotide variants (iSNV) distributed within the full genome in individual samples (Fig. 5 and Table S3). The highest diversity was observed in the lymph nodes on day 10 p.i. Three consensus sequence changes were detected in the spleen, cecum and lymph nodes (Fig. 5), two of which were nonsynonymous iSNVs (M73 V in prM and A227 V in E). The biological importance of these adaptive mutations in viral tropism and dissemination deserve further investigation.

### 3.4 ZIKV Induces Robust Humoral and Cellular Response

**Fig. 6:**
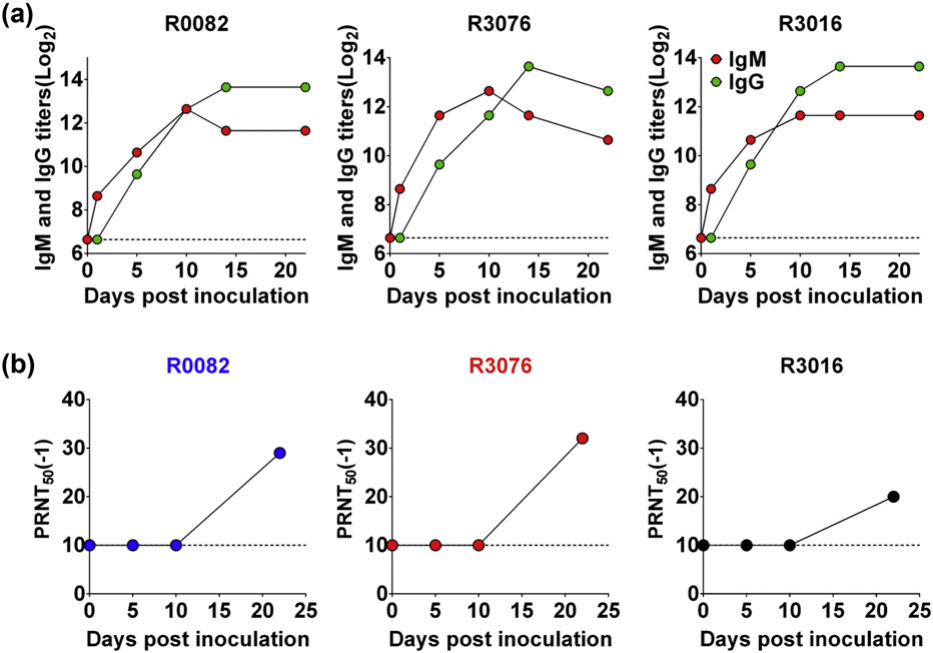
Humoral immune responses in ZIKV-infected rhesus monkeys. (a) Serum IgM and IgG antibodies against ZIKV were detected by ELISA using formaldehyde-inactivated ZIKV as an antigen source. The dotted lines represent the limits of detection of the ELISA assigned values of 100. (b) Neutralizing antibody titer of rhesus monkeys after s.c. challenge with ZIKV. Serial dilutions of inactivated serum were mixed with ZIKV. The concentration of infectious virus was determined using the plaque assay on BHK-21 cells. The endpoint neutralization titer was calculated according to the method of Reed and Muench. The dotted lines represent the limits of detection of the PRNT_50_ assigned values of 10.

Finally, to make sure whether the animal model described here could be used for vaccine efficacy tests, we further examined the ZIKV-specific humoral and cellular immune responses in all ZIKV-inoculated animals. As shown in Fig. 6a, ZIKV-specific IgM antibodies appeared on day 1 p.i., and rapidly increased on day 10 p.i. in all animals. The IgG kinetics was slightly delayed compared with IgM, and peaked on day 14 p.i. Importantly, all of the inoculated monkeys developed moderate level of neutralizing antibodies against ZIKV on day 22 p.i. (Fig. 6b).

Additionally, PBMC isolated from the infected animals were stimulated with ZIKV antigen and subjected to ELISPOT assay. Rapid increase in the production of IFN-γ, IL-2, and IL-10 were detected in all monkeys except one (R3016) on day 14 p.i. (Fig. S4). Collectively, these observations demonstrate that the subcutaneous inoculation of ZIKV induced humoral and cellular immune responses in rhesus monkeys, which paves the road to a ZIKV vaccine.

## 4 Discussion

The mechanism of ZIKV pathogenesis remains largely unclear. This is partly due to the lack of a robust animal model that recapitulates the clinical manifestations and disease kinetics as seen in ZIKV patients. Our results showed that rhesus macaques could be infected by the contemporary ZIKV strain that is circulating in south Americas. This non-human primate model described here partly recapitulates some clinical features and viral kinetics in ZIKV-infected patients, and therefore may serve as a model to study ZIKV disease and pathogenesis. Very recently, a rhesus macaque model of ZIKV infection was reported with a clinical strain isolated in French Polynesian in 2013 (Dudley et al., 2016). The 2013 ZIKV strain caused viremia and viral RNA shedding in urine and saliva, while viral shedding in lacrimal fluids was not determined. Specially, inappetence was seen in most ZIKV-infected animals (Dudley et al., 2016), and no other abnormal clinical sighs were noted in their study. In our study, four of five rhesus macaques developed fever upon ZIKV infection, that is directly associated with disease. The challenge dose used in Dudley's experiments was 100-fold lower than that in our study, which may account for the difference in clinical symptoms.

The viral shedding features in various body fluids in ZIKV-infected macaques correlated with the clinical findings from patients. The presence of high loads of viral RNA in these body fluids supports that besides blood, urine and saliva are now used for clinical diagnosis of ZIKV. Especially, our results demonstrate that viral RNA was abundant in lacrimal fluids in ZIKV-infected adult macaques, supporting the use of tears or lacrimal fluids for clinical diagnosis. ZIKV-associated damages have been documented in babies with microcephaly (de Paula Freitas et al., 2016, Ventura et al., 2016a, Ventura et al., 2016b), and conjunctivitis is also a common symptom in adult ZIKV patients (Dasgupta et al., 2016, Deng et al., 2016, Duffy et al., 2009). Whether the level of viral RNA in body fluids associated with disease severity remains unknown.

The in vivo replication kinetics of ZIKV in natural human infections remains elusive. The neurotropic nature of ZIKV has been evidenced by mouse experiments (Cugola et al., 2016) and clinical investigations (Carteaux et al., 2016). Our necropsy data from two animals demonstrate that the contemporary ZIKV strain can invade and replicate within the CNS system of macaques following s.c. inoculation, despite no neurological signs developed. However, the viral loads in CNS tissues are quite lower than other major target organs. In our experiments, few viral RNA or proteins was detected in the genital organs except bladder and testis on day 5 p.i. (Fig. 3), and no obvious pathological changes were seen in these organs. We are not able to collected semen samples due to technical reasons. High levels of positive- and negative-strand ZIKV RNAs were detectable in potential target organs at different stages of infection (day 5 or day 10 p.i.), and infectious ZIKV can be directly recovered from selected organs with high viral load. Unexpectedly, our results suggest that the parotid gland, besides intestinal tracts and spleen, represents potential major target tissues of ZIKV. The parotid gland serves as the viral replication site and exit portal for the highly neurotropic rabies virus into saliva (Boonsriroj et al., 2016). Although the biological importance of ZIKV replication in parotid gland remains unknown, a probable connection to the persistent excretion of viral RNA in saliva is highly suspected. Since our results are only from two animals dissected at different time, the in vivo kinetic of ZIKV replication deserves extensive investigation in the future.

In addition, the non-human primate model described here could be used to evaluate ZIKV vaccine or antiviral candidates. The reduction of body temperature increase, viremia, and excretion of viral RNA in various body fluids upon ZIKV infection could serve as endpoints for protection in vaccine or antiviral efficacy test. A large panel of ZIKV vaccine candidates is being developed in an expedited manner by using existing flavivirus vaccine platforms, e.g., chimeric live attenuated strains, killed virons, recombinant subunit viral proteins, subviral particles, or DNA plasmids, and clinical trials are highly expected in the near future (Weaver et al., 2016). The non-human primate model described here could serve as a gatekeeper for advancing vaccine candidates into clinics. Any candidate ZIKV vaccine is supposed to induce protective immune response and confer protection against contemporary ZIKV strain challenge in non-human primate model.

## Competing Interests

The authors have declared that no competing interests exist.

## Author Contributions

C.F.Q. and X.F.L. conceived and designed this study. X.F.L., H.L.D., H.J.W., Y.F.Q. and X.Y.H. performed the experiments. X.F.L., H.L.D., H.J.W., Y.Q.D., N.N.Z., Q.Y., H.Z., Z.Y.L., H.F., X.P.A., S.H.S. and C.F.Q. analyzed the data. F.C.Z., B.G., Y.Z.F., Y.G.T., G.F.G., W.C.C. and P.Y.S. contributed reagents and data analysis. X.F.L. and C.F.Q. wrote the paper. All authors edited and approved the manuscript.

## Acknowledgments & Funding

We thank the veterinarians from Laboratory Animal Center, Academy of Military Medical Science, for their excellent technical support; and Drs. Jing An, Zhiheng Xu, Xia Jia, and Bo Zhang for helpful discussion. This work was supported by the State Key Laboratory of Pathogen and Biosecurity (no. SKLPBS1601), the Guangzhou Science and Technology Program for Public Wellbeing (no. 201508020263, and no. 2014Y2-00550), the Beijing Nova Program (no. 2016110, and no. 2010B041), and the National Key Research and Development Project of China (no. 2016YFD0500304). CFQ was supported by the Excellent Young Scientist Program from the NSFC of China (no. 81522025) and the Newton Advanced Fellowship from the Academy of Medical Sciences, UK and the NSFC of China (No. 81661130162). PYS was partially supported by NIH grant AI087856, and a grant from Pan-American Health Organization and World Health Organization. All authors declared no conflicts of interest.

## Appendix A Supplementary Data

Supplementary material Image 1
