# Supplementary material for: Characterization of a 2016 Clinical Isolate of Zika Virus in Non-human Primates
Source: eBioMedicine. 2016 Sep 23;12:170–7. doi: 10.1016/j.ebiom.2016.09.022 (PMC5078627; doi:10.1016/j.ebiom.2016.09.022)
Supplement: Supplementary file 1 — Supplementary material [file mmc1.docx]

Supplemental information

**Characterization of a 2016 Clinical Isolate of Zika Virus in Non-human**

**Primates**

Xiao-Feng Li ^1, †^, Hao-Long Dong ^1, †^, Xing-Yao Huang ^1, †^, Ye-Feng Qiu ^2, †^, Hong-Jiang Wang ^1, &^, Yong-Qiang Deng ^1^, Na-Na Zhang ^1,3^, Qing Ye ^1^, Hui Zhao ^1^, Zhong-Yu Liu ^1^, Hang Fan ^1^, Xiao-Ping An ^1^, Shi-Hui Sun ^1^, Bo Gao ^1^, Yun-Zhi Fa ^2^, Yi-Gang Tong ^1^, Fu-Chun Zhang ^4^, George F. Gao ^5^, Wu-Chun Cao ^1^, Pei-Yong Shi ^6^, Cheng-Feng Qin^1, 3^ *

*Correspondence to: qincf@bmi.ac.cn


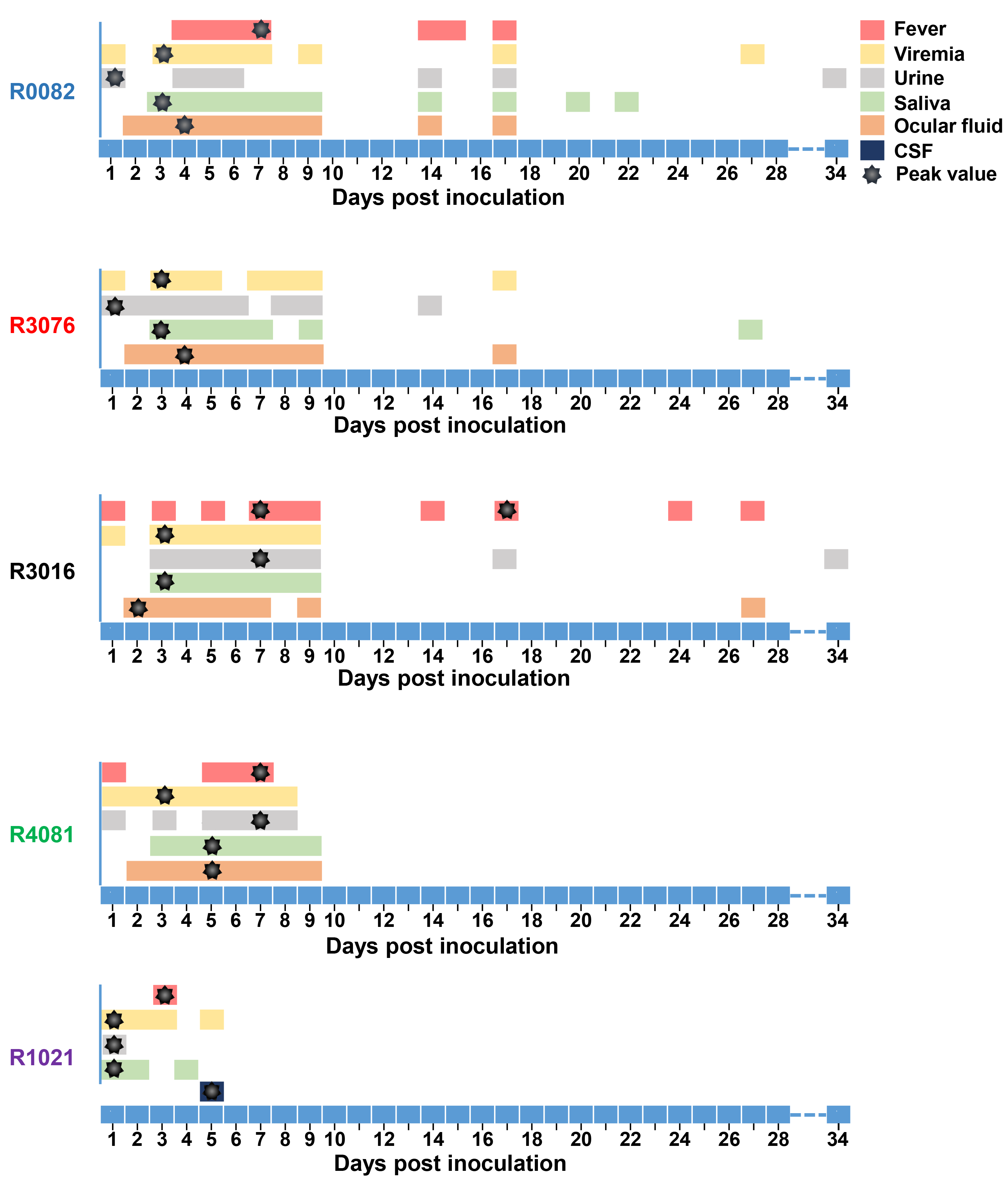


Figure S1. Timing of the onset and duration of clinical manifestations (fever and viremia) and excreted ZIKV RNA in major body fluids (urine, saliva and ocular fluid) in rhesus monkeys after s.c. inoculation.





**Figure S2.** **Forehead temperature of rhesus monkeys after s.c. challenge with ZIKV.** The dotted line indicates the value for determination of fever.


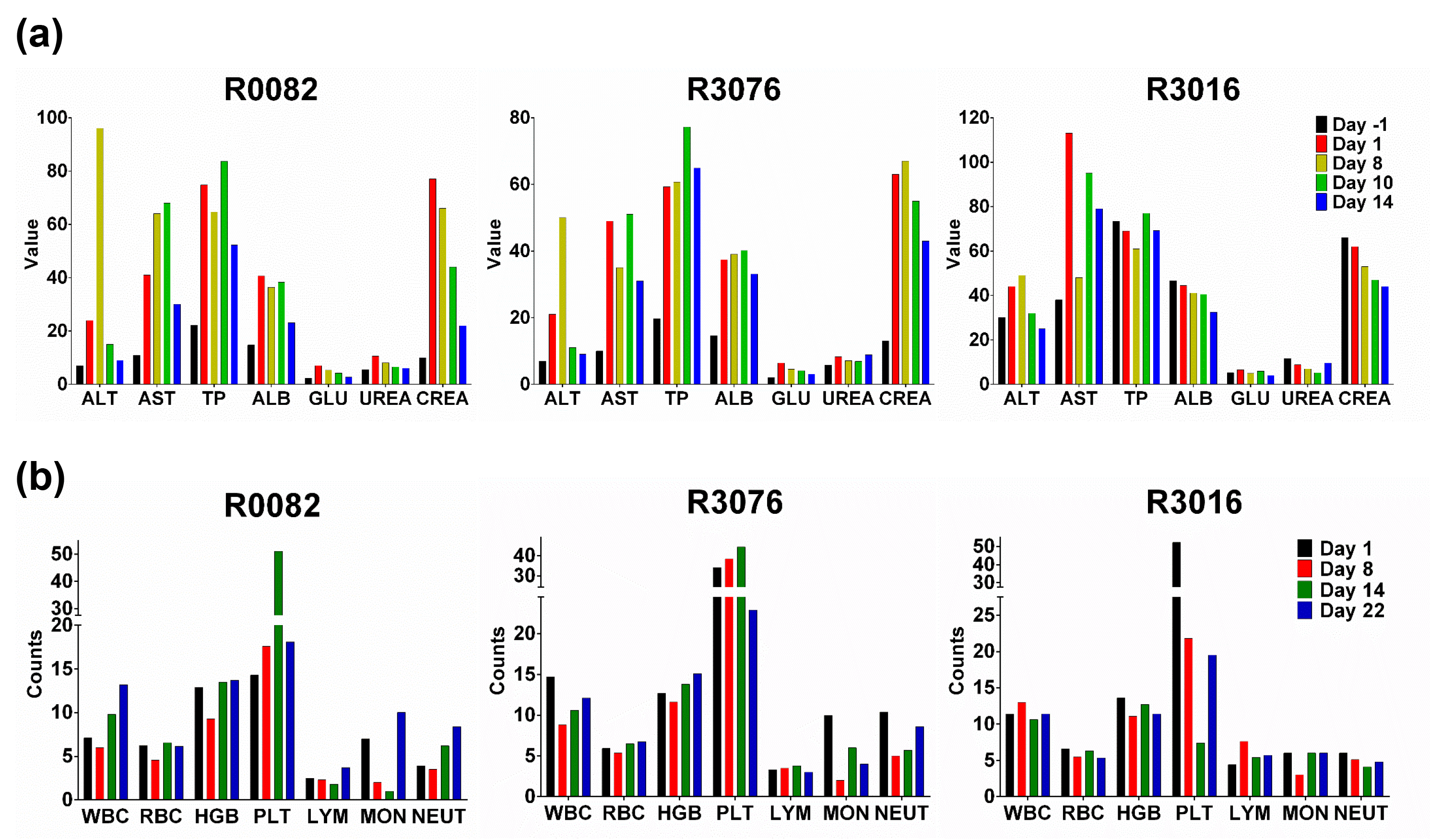


**Figure S3. Blood chemistry and blood cell counts for rhesus monkeys following s.c. challenge with ZIKV.** Animals were infected with 10^5^ PFU of ZIKV. (A) Chemistries (ALT, AST, TP ALB, GLU, UREA and CREA) were measured prior to infection and on 1, 8, 10 and 14 days p.i.. (B) Complete blood counts (WBC, RBC, HGB, PLT, LYM, MON and NEUT) were measured on days 1, 8, 14 and 22 p.i..


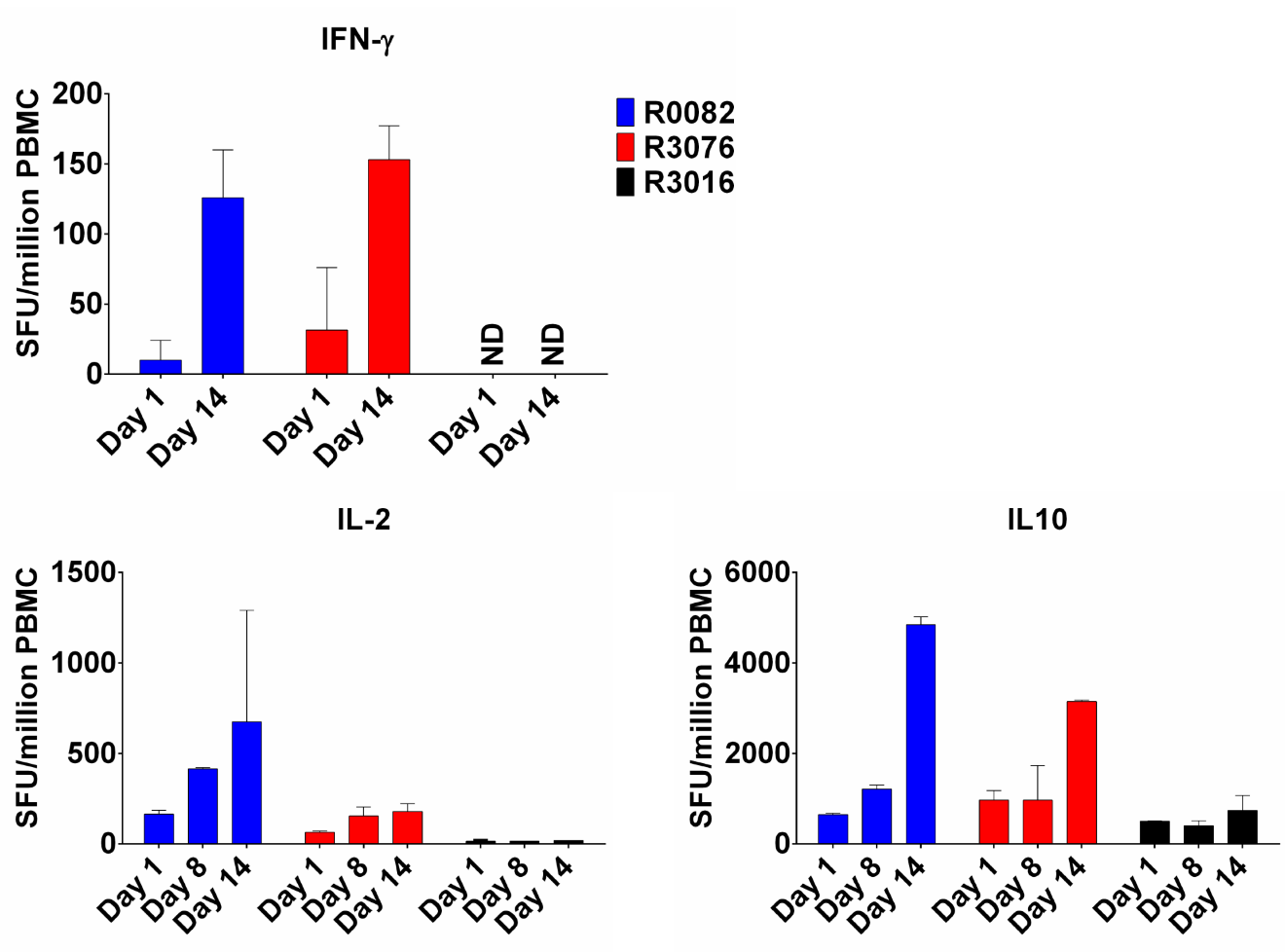


**Figure S4. Cellular immune responses in rhesus monkeys following s.c. ZIKV inoculation.** PBMCs of each inoculated animal were collected at days 1, 8, and 14 p.i. IFN-γ, IL-2 and IL-10 positive cells were determined by using ELSPOT.

**Table S1** **Oligonucleotides and fluorogenic probe used in qRT-PCR, qPCR and DNA sequencing**

| Name | Sequence (5’-3’) | Position* | Purpose |
| --- | --- | --- | --- |
| ZIKV-ASF | GGTCAGCGTCCTCTCTAATAAACG | 8507-8530 | qRT-PCR |
| ZIKV-ASR | GCACCCTAGTGTCCACTTTTTCC | 8631-8653 | qRT-PCR |
| ZIKV-Probe | FAM-AGCCATGACCGACACCACACCGT-BQ1 | 8586-8608 | qRT-PCR or qPCR |
| ZIKV-ASF-Tag | GGCAGTATCGTGAATTCGATGCgGTCAGCGTCCTCTCTAATAAACG | 8507-8530 | qPCR |
| ZIKV-ASR-Tag | GGCAGTATCGTGAATTCGATGCgCACCCTAGTGTCCACTTTTTCC | 8631-8653 | qPCR |
| Tag | GGCAGTATCGTGAATTCGATGC | NA^#^ | qPCR |
| ZIKV-F1 | ATGAAAAACCCAAAAAAGAAATCC | 1-24 | DNA sequencing |
| ZIKV-R1 | CCAAGTATATGACTTTTTGGCTC | 816-838 | DNA sequencing |
| ZIKV-F2 | CGCAAACCTGGTTGGAATCA | 692-711 | DNA sequencing |
| ZIKV-R2 | CCACGACAGTTTGCCTTTTGG | 1619-1639 | DNA sequencing |
| ZIKV-F3 | GGAAGCCTAGGACTTGATTGT | 1420-1440 | DNA sequencing |
| ZIKV-R3 | CGAGCACCCCACATCAGCAGAG | 2376-2397 | DNA sequencing |
| ZIKV-F4 | CAGCACCATTGGAAAAGCATTT | 2082-2103 | DNA sequencing |
| ZIKV-R4 | GAACCCATGATCCTCCACAAGAAAG | 2838-2862 | DNA sequencing |
| ZIKV-F5 | CGTCAGAGCAGCAAAGACAAAT | 2751-2772 | DNA sequencing |
| ZIKV-R5 | CACTGCCATTGATGTGCTTATGATGATC | 3531-3558 | DNA sequencing |
| ZIKV-F6 | GAAGGGCTGAAGAAGAGAATGACC | 3502-3525 | DNA sequencing |
| ZIKV-R6 | CCGCATCTTTTTCCCATGTGATGTC | 4288-4312 | DNA sequencing |
| ZIKV-F7 | GGGTTCGCCAAGGCAGATATAG | 4174-4195 | DNA sequencing |
| ZIKV-R7 | CTTCTTCTTCAGCATCGAAGGCTC | 5044-5067 | DNA sequencing |
| ZIKV-F8 | CCTAGACAAGTGTGGGAGAGTGATAG | 4923-4948 | DNA sequencing |
| ZIKV-R8 | GTTGGCGCCCATCTCTGAAATGTC | 5734-5757 | DNA sequencing |
| ZIKV-F9 | GGAACGGCAATGAGATCGCAGC | 5606-5627 | DNA sequencing |
| ZIKV-R9 | TGGCTTCCTGGAATCTCTCTGTCAT | 6406-6430 | DNA sequencing |
| ZIKV-F10 | CAAACCGAGGTGGATGGACGCCAG | 6276-6299 | DNA sequencing |
| ZIKV-R10 | TGGCCACTATTAGGGTCAGGGGTGTT | 7119-7144 | DNA sequencing |
| ZIKV-F11 | CCACCTCATACAACAACTACTC | 6983-7004 | DNA sequencing |
| ZIKV-R11 | GGGCTGCAGGTATCCCCGCT | 7760-7779 | DNA sequencing |
| ZIKV-F12 | CTCAAGGACGGTGTGGCAAC | 7690-7709 | DNA sequencing |
| ZIKV-R12 | CCTGACAACCCCGTTTATTAG | 8521-8541 | DNA sequencing |
| ZIKV-F13 | GGTAACCGCATTGAAAGGATCCGCAG | 8395-8420 | DNA sequencing |
| ZIKV-R13 | GTGTCCCAGCCAGCAGTGTCATC | 9160-9182 | DNA sequencing |
| ZIKV-F14 | GGAGAGAGAACTCAGGAGGTGGTGTTG | 9059-9085 | DNA sequencing |
| ZIKV-R14 | TTCTCCCTTTCCATGGATTGACC | 9950-9972 | DNA sequencing |
| ZIKV-F15 | GCCTAGCAAAATCATATGCGCAAAT | 9824-9848 | DNA sequencing |
| ZIKV-R15 | TTACAGCACTCCAGGTGTAGACCCT | 10248-10272 | DNA sequencing |

* Position numbers of matching nucleotides correspond to the ZIKV genome sequence (GenBank number KU820898)

^#^ NA, not applicable.

**Table S2 Viral genetic diversity in selected tissues of animals s.c. inoculated with ZIKV**

| Tested tissue | Days p.i. | Position* | Region | Nucleotide change | AA change | Variant frequency(%) |
| --- | --- | --- | --- | --- | --- | --- |
| Spleen | 5 | 142 | C-48 | T-> C | None | 9.5 |
|  |  | 529 | PrM-73 | A-> G | M->V | 5.9 |
|  |  | 2427 | NS1-15 | C->T | None | 7.1 |
|  |  | 3404 | NS1-341 | C->T | P->L | 5.1 |
|  |  | 4176 | NS2B-29 | G->T | None | 8.0 |
|  |  | 4726 | NS3-74 | C->T | Nonsense | 5.5 |
|  |  | 5623 | NS3-373 | G->A | A->T | 5.4 |
| Small intestine | 5 | 489 | PrM-59 | T->A | None | 14.6 |
|  |  | 3142 | NS1-254 | C->T | H->Y | 5.4 |
|  |  | 4659 | NS3-51 | C->T | None | 52.5 |
|  |  | 8235 | NS5-225 | G->A | None | 5.7 |
|  |  | 9783 | NS5-741 | C->T | None | 10.5 |
| Large intestine | 5 | 534 | PrM-74 | G->A | None | 6.7 |
|  |  | 3410 | NS1-343 | G->T | S->I | 7.2 |
|  |  | 8196 | NS5-212 | C->A | None | 5.5 |
|  |  | 9078 | NS5-506 | T->C | None | 7.6 |
|  |  | 9530 | NS5-657 | T->C | L->P | 6.3 |
| Cecum | 5 | 529 | PrM-73 | A-> G | M->V | 14.5 |
|  |  | 1550 | E-227 | C->T | A->V | 16.9 |
|  |  | 2406 | NS1-8 | C->T | None | 47.2 |
|  |  | 3726 | NS2A-96 | A-> G | None | 21.4 |
|  |  | 5682 | NS3-392 | G-> A | None | 23.4 |
|  |  | 5703 | NS3-399 | A-> G | None | 8.8 |
|  |  | 5713 | NS3-403 | T->C | W->R | 8.8 |
|  |  | 5746 | NS3-414 | A-> G | M->V | 11.8 |
|  |  | 7104 | NS4B-122 | C->T | None | 5.7 |
| Spleen | 10 | 888 | E-6 | C->T | None | 5.2 |
|  |  | 3174 | NS1-264 | G->T | None | 6.6 |
|  |  | 3259 | NS1-293 | A->G | T->A | 6.3 |
|  |  | 3411 | NS1-343 | C->T | None | 9.0 |
|  |  | 4176 | NS2B-29 | G->T | None | 11.5 |
|  |  | 4674 | NS3-56 | C->T | None | 5.8 |
|  |  | 5893 | NS3-463 | A->G | N->D | 7.7 |
|  |  | 6441 | NS4A-28 | C->T | None | 5.2 |
|  |  | 7253 | NS4B-172 | T->C | V->A | 6.1 |
| Lymphonodus | 10 | 326 | PrM-5 | G-> A | S->N | 17.2 |
|  |  | 399 | PrM-29 | T->C | None | 13.3 |
|  |  | 617 | PrM-102 | A->G | K->R | 6.0 |
|  |  | 660 | PrM-116 | T->C | None | 10.7 |
|  |  | 699 | PrM-129 | C->T | None | 10.1 |
|  |  | 717 | PrM-135 | A->G | None | 9.2 |
|  |  | 789 | PrM-159 | T->C | None | 6.2 |
|  |  | 864 | PrM-184 | A->G | None | 6.1 |
|  |  | 1254 | E-128 | A->G | None | 8.7 |
|  |  | 1368 | E-166 | A->G | None | 8.3 |
|  |  | 1550 | E-227 | C->T | A->V | 5.2 |
|  |  | 1944 | E-358 | G->A | None | 6.5 |
|  |  | 2322 | E-484 | T->C | None | 11.4 |
|  |  | 2442 | NS1-20 | T->C | None | 12.1 |
|  |  | 4176 | NS2B-29 | G->T | None | 24.8 |
|  |  | 4210 | NS2B-41 | A->G | M->V | 6.0 |
|  |  | 7221 | NS4B-161 | A->G | None | 8.1 |
|  |  | 8055 | NS5-165 | C->T | None | 12.6 |
|  |  | 8100 | NS5-180 | C->T | None | 12.2 |
|  |  | 8115 | NS5-185 | C->T | None | 12.3 |
|  |  | 8922 | NS5-454 | C->T | None | 5.2 |

*Position numbers of matching nucleotides correspond to the ZIKV genome sequence (GenBank accession no: KU820898)

| Days post inoculation | Sample | Positive-strand RNA | | Negative-strand RNA | |
| --- | --- | --- | --- | --- | --- |
|  |  | Ct value | RNA copies/g(Log_10_) | Ct value | RNA copies/g(Log_10_) |
| 5 | Parotid gland | 30.16 | 5.51257944 | 34.8 | 4.18832344 |
|  | Spleen | 29.73 | 6.167725504 | 34.74 | 4.737871504 |
|  | Large intestine | 29.8 | 6.181171259 | 34.46 | 4.851207259 |
|  | Small intestine | 31.04 | 5.526245263 | 31.7 | 5.036851268 |
| 10 | Spleen | 29.98 | 6.06534127 | 34.06 | 4.90090927 |
|  | Lymphonodus | 31.21 | 5.553447977 | 35.34 | 4.374745977 |

Table S3 Quantification of specific strand of viral RNA in organs of monkeys infected with ZIKV
